# Biologic Therapies and Autoimmune Phenomena

**DOI:** 10.31138/mjr.32.2.96

**Published:** 2021-06-30

**Authors:** Alexandros A. Drosos, Eleftherios Pelechas, Evripidis Kaltsonoudis, Theodora E. Markatseli, Paraskevi V. Voulgari

**Affiliations:** Rheumatology Clinic, Department of Internal Medicine, Medical School, University of Ioannina, Ioannina, Greece

**Keywords:** Biologic agents, autoimmune phenomena, paradoxical inflammation, adverse events, treatment

## Abstract

The use of biologic medications has represented a great advancement in the treatment of autoimmune rheumatic diseases. Despite their excellent efficacy, during the last years, a growing number of reports of autoimmune phenomena and paradoxical inflammation has emerged. These phenomena may range from the discovery of an isolated autoantibody to full-blown autoimmune diseases, organ-specific and systemic. This review has been carried out in order to underline the multitude of the potential adverse manifestations from the use of biologic medications. Thus, early recognition of specific types of autoimmune phenomena is an imperative for the physicians allowing them to have an accurate diagnosis and treatment.

## INTRODUCTION

Our immune system has evolved over millions of years, and has developed well-organized and sophisticated defence mechanisms to protect the host against infections, or cancer while maintaining homeostasis through negative regulation of cell activation. The principal cells of immune system are T and B lymphocytes, antigen presenting cells, and effector cells. These cells are concentrated in anatomical discreet sites which are called lymphoid organs, and are also present in the peripheral blood. The cells of the immune system interact with one another and host cells through soluble mediators (proteins), produced by various cells, named cytokines.^1^ Autoimmune rheumatic diseases (ARD) are characterized by dysregulation of the immune system with aberrant activation of lymphocytes and macrophages in the target organs, as well as increased production of pro-inflammatory cytokines like tumour necrosis factor a (TNFα), interleukin (IL)-1, IL-6, IL-17, interferons α, β (INF), which are found in the tissue of inflammation and in peripheral blood.^2^ To this end, during the last two decades, biologic agents targeting cytokines or T and B cells have been developed and used for the treatment of ARDs, which have revolutionised the management of these disorders.^3^ Biological drugs targeting compounds of the immune system have been used for the treatment of rheumatoid arthritis (RA), psoriasis, psoriatic arthritis (PsA), ankylosing spondylitis (AS), inflammatory bowel diseases (IBD), systemic lupus erythematosus (SLE), and vasculitis.^4^

However, by blocking the cytokines or lymphocytes of the immune system, adverse events may occur. Among them mycobacterial, bacterial, viral or other infections have been reported,^5,6^ as well as several autoimmune phenomena. The autoimmune phenomena range from an isolated presence of an antibody, mainly antinuclear antibody (ANA), to full-blown autoimmune diseases.^7^

In this review we will discuss the autoimmune phenomena related mainly with the use of the TNFα inhibitors (TNFαi), which are the most widely used among biological agents.^8,9^

## BIOLOGICAL AGENTS

Specific biologic agents against cytokines and lymphocytes have been developed and approved for the treatment of ARDs. The most commonly used are the TNFαi which include: the monoclonal antibodies (mAb) adalimumab (ADA), infliximab (INF), golimumab (GOL), the soluble TNF receptor IgG Fc fusion protein etanercept (ETN), the pegylated antibody fragment certolizumab (CTZ),^10–14^ and TNFαi biosimilars.^15,16^ Other cytokine inhibitors comprise the mAb tocilizumab (TCZ) and sarilumab (SAR), which are IL-6 receptor antagonists.^17,18^ Anakinra is an IL-1 receptor antagonist while, secukinumab is an IL-17A mAb, and ustekinumab an IL-12/IL-23 mAb.^10^ **Table 1** depicts the biologic agents used in the treatment of ARD.

**Table 1.** Biological agents for the treatment of autoimmune rheumatic diseases.

**Target**	**Agent**	**Structure**	**Indications**

Cytokines TNFα	Adalimumab	• Fully human-TNFα mAb	• RA, PsA, AS, PsO, JIA, CD, UC
	Golimumab	• Fully human-TNFα mAb	• RA, PsA, PsO, AS, UC
	Infliximab	• Chimeric –TNFα mAb	• RA, PsA, AS, PsO, UC, CD
	Etanercept	• Soluble TNF receptor IgG Fc fusion protein	• RA, PsA, AS, PsO, JIA
	Certolizumab Peg	• Humanized Fab’ Fragment linked to pegylated molecule	• RA, PsA, AS, PsO, CD

IL-1 receptor	Anakinra	Recombinant IL-1 receptor antagonist	RA, CAPS

IL-6 receptor	• Tocilizumab •Sarilumab	Humanized anti IL-6 receptor mAb	RA, JIA, TA

IL-12/IL-23	Ustekinumab	Fully human anti IL-12/IL-23 mAb	PsA, PsO

IL-17	Secukinumab	Fully human anti-17A mAb	PsA, AS, PsO

Lymhocyte • T-cell CD28	• Abatacept	CTLA-4 IgG Fc fusion protein	RA, JIA
• B cell CD20	• Rituximab	Chimeric anti-CD20 mAb	SLE
BAFF	• Belimumab	Fully human mAb for solute BAFF	SLE

Biosimilars • TNFα • CD20	• Adalimumab • Etanercept • Rituximab		

AS: Ankylosing Spondylitis; BAFF: B-cell activation factor; CAPS: cryopyrin-associated periodic syndrome; CD: Crohn’s disease; CLL: chronic lymphocytic leukaemia; CTLA4: cytotoxic T-lymphocyte antigen 4; Fab: Fragment antigen binding; GPA: granulomatosis with polyangiitis; JIA: juvenile idiopathic arthritis; MPA: microscopic polyangiitis; mAb: monoclonal antibody; NHL : non-Hodgkin lymphoma; PsA: psoriatic arthritis; Pso: psoriasis; RA: rheumatoid arthritis; SLE: systemic lupus erythematosus; TA: temporal arteritis; UC: ulcerative colitis.

## AUTOIMMUNE PHENOMENA AND SYSTEMIC DISEASES

Autoimmune phenomena associated with biological therapies usually manifest during the first months of treatment (1–8 months), but can also manifest after years in a few patients. The autoimmune phenomena and clinical manifestations range from an isolated presence of an antibody, mostly ANA, or double stranded DNA (ds-DNA), to full blown autoimmune diseases, organ specific and systemic.^19–21^

**Figure 1.**
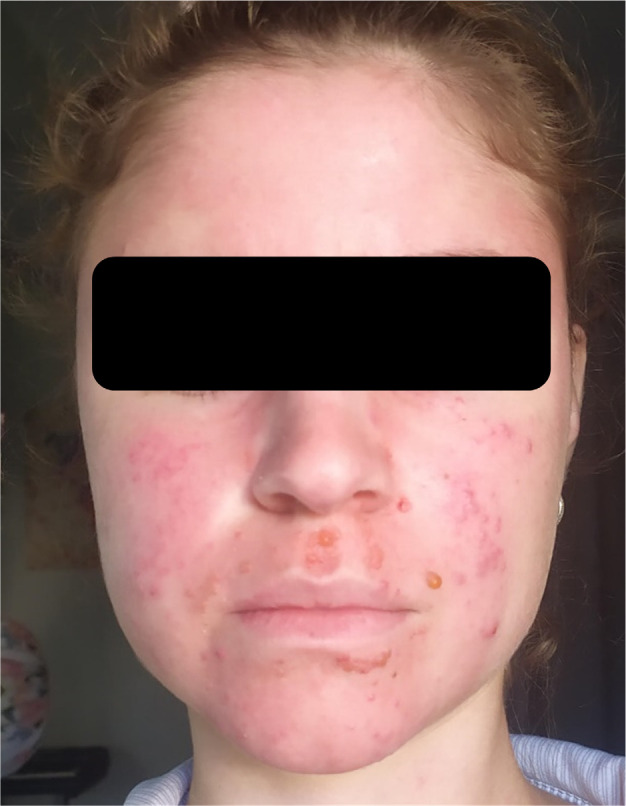
A 26-year-old woman with seropositive rheumatoid arthritis refractory to methotrexate received adalimumab 40mg every 14 days subcutaneously. She responded very well, but 6 months later she developed erythematous eruptions affecting her face in a butterfly distribution and with blister formation.

The organ specific diseases include: uveitis, multiple sclerosis (MS) like lesions, myelitis, optic neuritis, inter-stitial lung disease (ILD) and others. Systemic diseases comprise drug-induced lupus (DIL), systemic vasculitis, antiphospholipid syndrome (APS), inflammatory myopathies, and others.^22–26^ The occurrence of antibodies manifested during the use of biologic agents varies considerably among studies. ANAs have been reported to occur from 40–100%,^20,27^ dsDNA from 11–62%,21,25 and antiphospholipid antibodies (APLs) between 2–12%,^21,25^ while other antibodies like Sm, Ro (SSA), La (SSB) have been reported less frequently.^22,23^ The clinical manifestations of DIL due to TNFαi comprise arthritis, photosensitivity, skin manifestations (malar rash, discoid, annular and psoriasiform lesions) (**Figure 1**), oral ulcers, myalgias, while kidney disease and central nervous system (CNS) involvement are uncommon. However, patients with severe lupus present with a plethora of antibodies such as ds-DNA, Ro (SSA) or La (SSB) and low complement levels.^22–26^

The mechanisms to explain the production of antibodies and DIL development during TNFαi are not well understood. However, one hypothesis is that TNFαi can induce cell apoptosis, generation of antigenic material, and ultimately antibody formation with lupus manifestations.^28^ Another theory is that TNFαi can interfere with Th1/Th2 immune response in which there is a Th1 suppression in favor of Th2 response with the production of IFNa and β which can evolve in lupus pathogenesis.^7,29,30^ Another speculation is that a latent infection in patients treated with TNFαi may trigger the formation of antibodies and DIL manifestations.^7,29,30^ Regarding the treatment in these patients, TNFαi discontinuation is an imperative. Furthermore, local calcineurin inhibitors, with or without small doses of prednisone for skin manifestations are sufficient, and patients respond well until complete resolution. However, even rare, in patients who develop severe lupus manifestations, such as lupus nephritis or CNS manifestations, high doses of prednisone and immunosuppressive drugs are required.^22,24,31^

## PARADOXICAL INFLAMMATION

**Figure 2.**
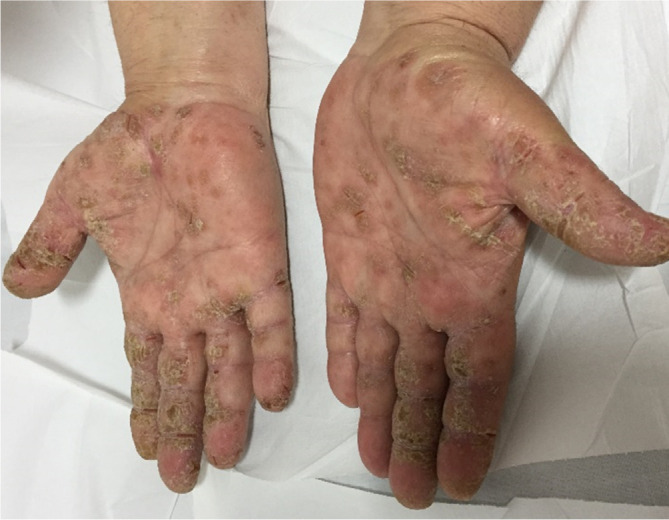
A 62-year-old man with seropositive rheumatoid arthritis refractory to methotrexate and leflunomide was treated with SB4, an etanercept biosimilar 50mg/week subcutaneously. After 3 months he developed psoriasiform eruptions affecting the palms of the hands.

The most common autoimmune phenomenon related to biological therapies, especially with the use of TNFαi is the paradoxical inflammation. This is an intriguing manifestation that can be presented with the same clinical features for which TNFαi are used to treat symptoms of RA, PsA, psoriasis, and IBD (inflammatory bowel disease). Thus, the paradoxical inflammation phenomenon can be presented as arthritis, uveitis, psoriasis, colitis, and other manifestations.^32–35^ Among them, the most common manifestation is that of psoriatic skin lesions.^36–38^ The prevalence of psoriatic lesions following TNFαi therapy ranges between 0.6–5.3% in RA patients, approximately 4% in SpA and 1.6–10% in IBD (inflammatory bowel disease) patients.^32–35,39^ The regions involved in this paradoxical inflammation are mainly the palmoplantar skin and the scalp, however other regions may be affected.^36,37^ Some investigators argued whether TNFαi induced psoriasis is a true manifestation, or is an occult clinical feature which is manifested later on, during the disease course. It is of interest that TNFαi biosimilars manifested the same paradoxical inflammation of psoriasis, as reported recently by Pelechas et al.^40^ (**Figure 2**) Indeed, he described an RA patient who was treated with SB4, an ETN biosimilar, and developed psoriatic skin lesions affecting the palms of the hands, 8 months after SB4 therapy initiation. The mechanisms responsible for this autoimmune phenomenon has not been elucidated. However, it is proposed that TNFαi may cause a cytokine shift of TNFα and IFNα. It is known that plasmacytoid cells secrete a large amount of IFNα which accumulate in the skin in the early phase of the disease process, and contribute to psoriasis pathogenesis.^7^ On the other side, TNFα prevents the generation of plasmacytoid dendritic cells and downregulates the production of IFNα. Thus, neutralization of TNFα following TNFαi increased the production of IFΝα by plasmacytoid dendritic cells with the development of skin psoriasis.^41–43^ In addition, other biological therapies can also induce psoriasis. Some case reports and case series of RA and SLE patients treated with rituximab (RTX) developed psoriatic skin lesions.^44–46^ It is postulated that B-cell population depletion from RTX may lead to upregulation of T-cell population and the development of psoriasis.^47^

**Figure 3.**
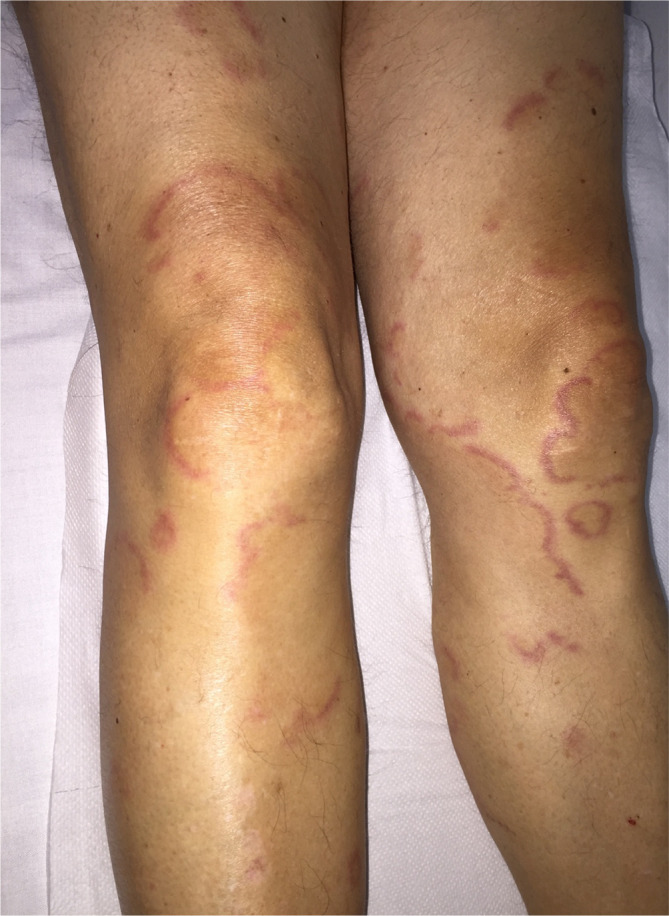
A 65-year-old man with seropositive rheumatoid arthritis refractory to methotrexate and infliximab. He was treated with tocilizumab 162mg/week subcutaneously. He responded very well to the above treatment but eight months later he developed polycyclic skin rashes affecting the lower extremities. The histological picture was compatible with granuloma annulare.

Another well-documented paradoxical manifestation due to TNFαi is granuloma annulare (GA) formation. Davos et al. describes the first case of GA development in a patient with PsA treated with INF.^48^ Subsequently, 9 cases of well documented GA have been reported by Voulgari et al. in RA patients,^49^ while 2 other cases have been described using secukinumab for PsA and psoriasis, respectively.^50,51^ Furthermore, a new case of GA in RA patient treated with tocilizumab has been described recently.^52^ (**Figure 3**) The postulated mechanisms underlying this paradoxical manifestation propose that cytokine inhibitors may cause T-cell upregulation with subsequent macrophage activation leading to granuloma formation.^49–52^ Finally, many other autoimmune skin manifestations following TNFαi have been described such as: erythema multiforme, pustular folliculitis, skin vasculitis, vitiligo, and others.^38,53^

**Figure 4.**
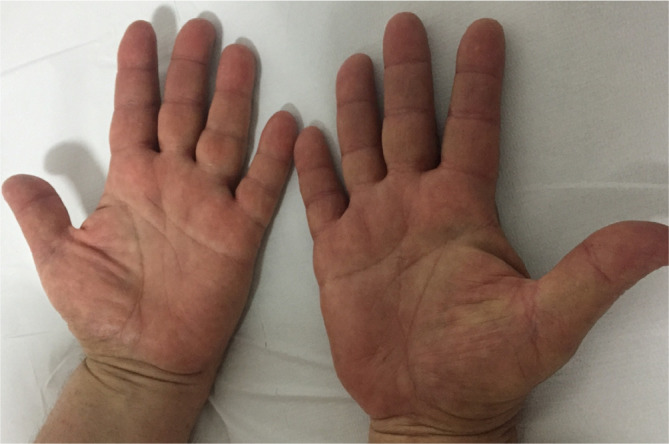
The same patient as in Figure 2, two months later after discontinuation of SB4 receiving small doses of prednisone. Note the complete resolution of the psoriasiform skin rashes.

**Figure 5.**
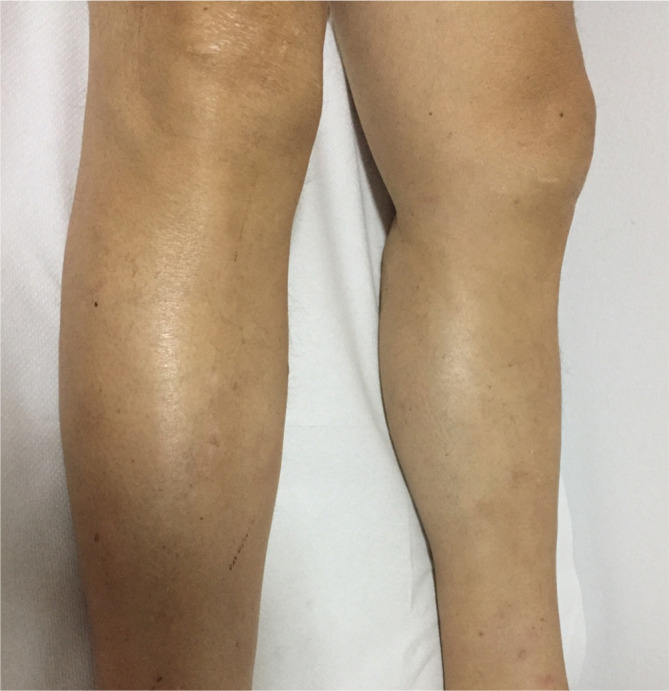
The same patient as in Figure 3, three months later after tocilizumab discontinuation receiving prednisone 10mg/day. Note the complete resolution of granuloma annulare eruptions.

As far as it concerns the treatment, cytokine inhibitors discontinuation may be sufficient in mild cases, however topical steroids and small doses of prednisone may be required in same cases. Usually, the skin manifestations subside with complete resolution after 2 months of biological agent’s discontinuation (**Figures 4 and 5**).

## NEUROLOGICAL MANIFESTATIONS (NM)

Neurological manifestations (NM) in patients receiving biologic agents have been described in case reports, case series, cross sectional and prospective observational studies from institutions and registries. Demyelinating disorders (DD), MS-like lesions, optic neuritis, myelitis, peripheral neuropathies, and others have been described in post-marketing surveillance mainly during TNFαi and RTX.^54–62^ The prevalence range from 0.05–0.2%.^25^ However, in one prospective study the estimated prevalence was 4%.^63^ Indeed, in this study 36 patients with RA and 41 with SpA (24 PsA, 17 AS) who were eligible for TNFαi therapy were investigated. Before treatment, all patients had a complete physical and neurological evaluation, and all had brain and cervical spine magnetic resonance imaging (MRI) and neurophysiological studies. Two patients without any objective clinical manifestation presented in their MRI findings compatible with MS-like lesions, and did not receive TNFαi therapy. These patients can be classified as having radiological isolated syndrome (RIS), which is considered a preclinical finding of MS. Finally, a total of 75 patients received TNFαi therapy. During follow-up (16–25 months) 3 patients developed NM. A patient with PsA who switched from ETN to INF developed clinical and imaging features of MS-like lesions after 8 months of INF therapy. One of the patients with RA treated with ADA, 8 months later developed optic neuritis, while another patient with AS and Crohn’s disease after 25 months of INF treatment developed peripheral sensorimotor polyneuropathy.^63^ All patients discontinued ant-TNFα therapy, and the clinical features disappeared two months later. Kopp et al. in a prospective observational study in patients with RA and SpA receiving TNFαi, found an increased risk of NM in SpA patients, as compared to controls without receiving TNFαi. On the other hand, they found no consistent and significant risk of NM after TNFαi in RA patients. They concluded that the risk of NM following TNFαi is disease-dependent and not agent-dependent.^64^ However, new autoimmune NM have been described in RA patients. Indeed, two patients one receiving ETN^65^ and the other ADA^66^ developed myasthenia Gravis. All the above indicate that NM following TNFαi therapy are drug-dependent and not disease-dependent. This means that it is a class effect phenomenon.^67^ The mechanisms responsible for the development of NM after TNFαi are unclear. However, some hypothesis propose that 1) TNFαi increase the autoreactive T-cells in the peripheral blood, which can penetrate into CNS causing MS-like lesions; 2) TNFαi may cause downregulation of TNF receptors 2 (TNFR2) in the brain, necessary for the proliferation of oligodendrocytes and damage repair; 3) downregulation of IL-10 and upregulation of IL-12 and IFNγ responsible for DD process; and 4) finally, a latent infection may be critical to initiate a DD process.^68^

In addition, treatment with biologic agents induced another DD known as progressive multifocal leukoencephalopathy (PML). This is a rare, but sometimes fatal DD of CNS, caused by the reactivation of John Cunningham virus (JCV). The incidence of PML development in patients TNFαi is very low. Some case reports have been published in this direction.^69^ However, efalizumab, a mAb against leucocyte function activation protein 1 (LFA1), that had been used for the treatment of plaque psoriasis since 2003, has been reported to cause PML.^70,71^ Thus, the authorities withdraw the drug from the market in 2009. On the other hand, RTX has been also associated with PML development. PML is a well-described pathology in oncology patients receiving RTX for non-Hodgkin lymphoma and chronic lymphocytic leukaemia.^60,61^ In the rheumatology setting, PML has been described to occur initially in patients with SLE and later on in RA patients and in patients with vasculitis receiving RTX. Between 2006–2015 approximately 351,396 RA patients have been treated with RTX and 9 cases with PML were identified giving a rate of 2.56/100,000, while only 2 cases of PML in patients with vasculitis have been reported since 2015.^60,61^ Treatment requires discontinuation of RTX, and steroids with or without other immunosuppressive drugs.

## OTHER AUTOIMMUNE PHENOMENA

Other autoimmune diseases, mainly organ specific following biologic agents’ therapy have been rarely reported. Inflammatory myopathies, ILD, inflammatory ocular diseases, IBD, sarcoidosis, and others are among them.^25,72–75^

## CONCLUSIONS

The use of biologic therapies has revolutionized the treatment of ARD. However, the use of these agents may affect the normal immune function and its response, leading to the development of many autoimmune phenomena and diseases. These phenomena range from isolated discovery of an autoantibody, to full-blown autoimmune diseases, organ specific, and systemic. This review has been carried out in order to underline the multitude of the potential adverse manifestations from the use of biologic agents. Early recognition of the specific types of autoimmune phenomena is an imperative for physicians. They must have the opportunity to diagnose and treat them appropriately early on their development avoiding misinterpretations and misdiagnoses that may exhaust patients with unnecessary laboratory tests and visits to different specialists. Thus, close follow-up and monitoring is mandatory.

## TAKE-HOME NOTES

The use of biologic agents has revolutionized the treatment of ARD. However, their use may alter the immune system function and its normal responses. A large number of clinical and laboratory autoimmune phenomena have been emerged ranging from an asymptomatic immunological alteration to organ specific and systemic diseases.

A careful and minute past medical and family history of pre-existing clinical features suggestive of an underlying autoimmune disease, followed by a detailed clinical and immunological evaluation before the initiation of biological therapy is mandatory.

Recognition of the specific types of autoimmune phenomena and diseases will allow physicians to have an accurate diagnosis and treatment plan. Thus, physicians dealing with patients treated with biological therapies should be aware of the possible development of several autoimmune phenomena, therefore close follow-up and monitoring is essential.

Treatment requires cessation of biologic agents, which probably may be sufficient in mild cases, but in cases of systemic manifestations, small or high doses of prednisone with or without immunosuppressive drugs are required. Usually, the prognosis and outcome are good.
